# Genetic and molecular biology of autism spectrum disorder among Middle East population: a review

**DOI:** 10.1186/s40246-021-00319-2

**Published:** 2021-03-12

**Authors:** Zahra Rahmani, Mohammad Reza Fayyazi Bordbar, Mohsen Dibaj, Maliheh Alimardani, Meysam Moghbeli

**Affiliations:** 1 Department of Medical Genetics, Golestan University of Medical Sciences, Gorgan, Iran; 2 Psychiatry and Behavioral Sciences Research Center, Mashhad University of Medical Sciences, Mashhad, Iran; 3 Department of Biological Sciences, School of Natural Sciences, University of Tabriz, Tabriz, Iran; 4 Department of Medical Genetics and Molecular Medicine, School of Medicine, Mashhad University of Medical Sciences, Mashhad, Iran; 5 Student Research Committee, Faculty of Medicine, Mashhad University of Medical Sciences, Mashhad, Iran

**Keywords:** Autism, Genetic, Risk factor, Diagnosis, Prognosis, Middle East

## Abstract

**Background:**

Autism spectrum disorder (ASD) is a neurodevelopmental disease, characterized by impaired social communication, executive dysfunction, and abnormal perceptual processing. It is more frequent among males. All of these clinical manifestations are associated with atypical neural development. Various genetic and environmental risk factors are involved in the etiology of autism. Genetic assessment is essential for the early detection and intervention which can improve social communications and reduce abnormal behaviors. Although, there is a noticeable ASD incidence in Middle East countries, there is still a lack of knowledge about the genetic and molecular biology of ASD among this population to introduce efficient diagnostic and prognostic methods.

**Main body:**

In the present review, we have summarized all of the genes which have been associated with ASD progression among Middle East population. We have also categorized the reported genes based on their cell and molecular functions.

**Conclusions:**

This review clarifies the genetic and molecular biology of ASD among Middle East population and paves the way of introducing an efficient population based panel of genetic markers for the early detection and management of ASD in Middle East countries.

## Background

**Fig. 1:**
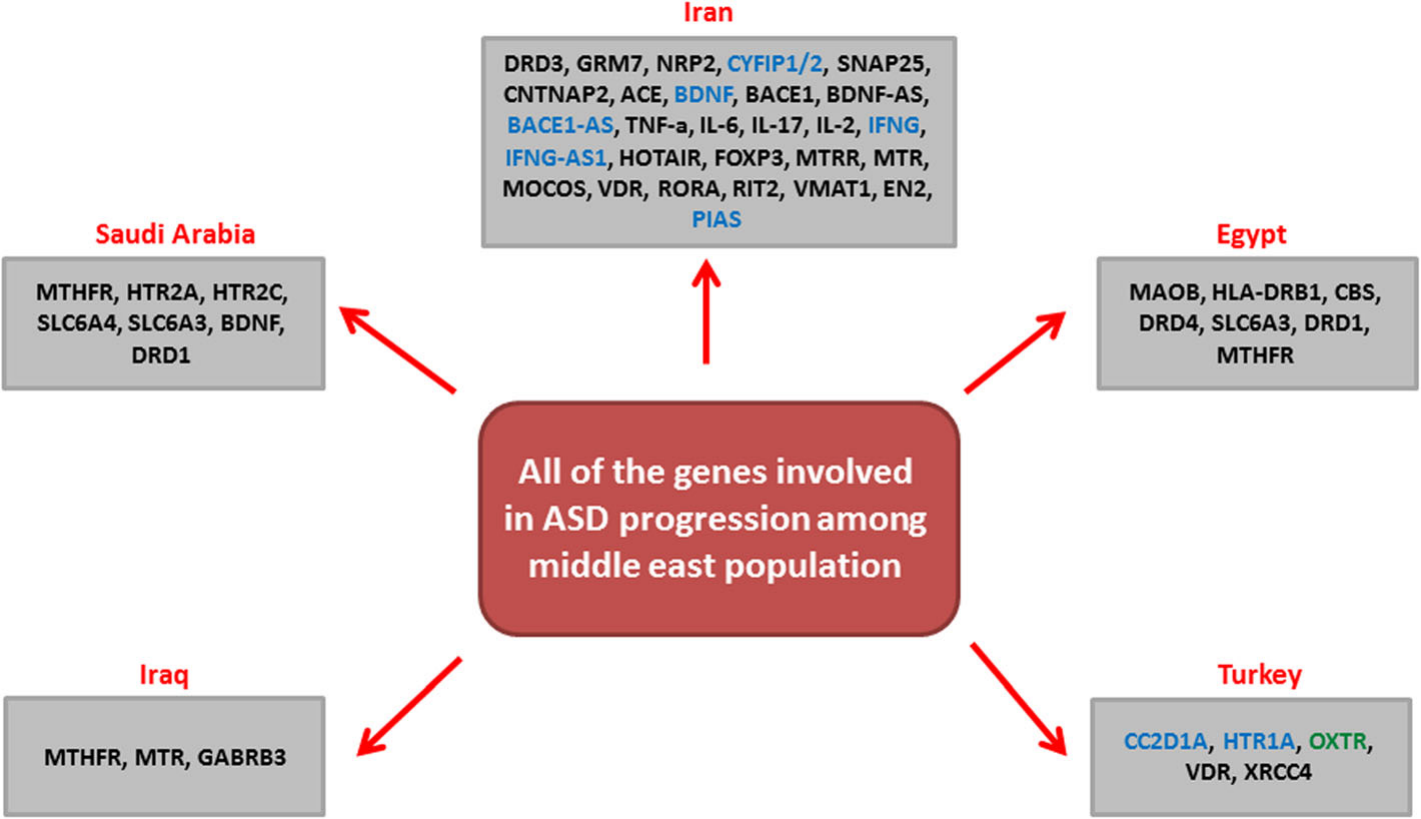
All of the genetic aberrations involved in ASD progression among Middle East population. Blue, green, and black colors refer to the aberrant expression, promoter methylation, and polymorphisms, respectively

Autism spectrum disorder (ASD) is a neurodevelopmental disease with disrupted social and emotional communication, learning problems, anxiety, epilepsy, language defect, and restrictive behaviors [1, 2]. All of these clinical manifestations can be related to the reduced size of cells in the hippocampus and limbic system and elevated number prefrontal cortex neurons [3]. ASD patients have also unusual temporal and frontal lobe development and less amygdala volume and gray matter in MRI results which indicated limited brain development in these patients [4]. The prevalence of ASD is approximately 2.47%, 0.06%, and 0.36% in the USA, Iran, and Asia, respectively [1, 5]. The range of ASD prevalence has been reported between 0.14 and 2.9 percentages in Persian gulf populations [6]. Various genetic and environmental factors are involved in ASD progression. Maternal fever and infections, zinc deficiency, folate prenatal intake, and air pollution are among the important environmental risk factors of ASD [7]. The high maternal and paternal age and mitochondrial dysfunction are also the risk factors of ASD [8, 9]. About 800 genes involved in synapse function, chromatin modification, and cortical development are associated with autism [10]. NL3, HOXA1, ANK2 MECP2, and ITGB3 mutations were associated with brain morphology and function. CNTNAP2 has a key role in enhanced frontal lobe connectivity and activation of AKT/mTOR pathway [1]. Moreover, 10% of all ASD cases are related with other genetic disorders such as fragile X, Noonan, and Rett syndromes [11]. Single nucleotide polymorphisms (SNP), copy number variations, and epigenetic modifications are the most frequent genetic alterations in ASD patients [12, 13]. The most significant susceptibility locus associated with ASD is located on chromosome 7, especially 7q31, which includes FOXP2, RAY1/ST7, and IMMP2L genes [14]. Moreover, epigenetic deregulations such as DNA methylation and histone modification have pivotal role during ASD progression [11]. Since the recurrence rate of ASD is higher in families with one affected child, genetic counseling plays an important role in prevention of other affected child birth. Early diagnosis can also improve the quality of life in ASD children. Because of heterogeneity of ASD, whole exome sequencing, ASD/ID panel, and chromosomal microarray analysis have been suggested for ASD diagnosis [9]. Behavioral therapy techniques such as peer feedback and video modeling are suggested to improve the quality of life and learning and social relationship in these patients [15]. Psychiatrists routinely use medications such as anticonvulsants, alpha-2 agonists, antidepressants, and antipsychotics for treatment of patients with autism spectrum [16]. Regarding the rising trend of ASD incidences in Middle East countries, it is required to assess the molecular biology of ASD in this area. In present review, we have summarized all of the significant genetic variations associated with ASD in Middle East population for the first time in the world (Fig. 1, Table 1). PubMed, Scopus, Embase, and Web of Science were used for the data collection. The search strategy in PubMed was based on MeSH: “Autism Spectrum Disorder and Genetic” in Middle East countries until the November of 2020. This review clarifies the genetic and molecular biology of ASD and paves the way of introducing a population-based panel of genetic markers for the early detection and better management of ASD among Middle Eastern population.

**Table 1:** All of the genes which have been involved in ASD progression in Middle East population

Study	Year	Gene	Country	Population	Results
Receptors and neurotransmitters					
Firouzabadi et al. [17]	2017	DRD3	Iran	56 patients controls	Polymorphisms were correlated with response to risperidone in autism patients.
Noroozi et al. [18]	2016	GRM7	Iran	518 patients 472 controls	Polymorphisms were correlated with the risk of ASD.
Elhawary et al. [19]	2019	HTR2A HTR2C SLC6A4 BDNF	Saudi Arabia	110 patients 102 controls	Polymorphisms were correlated with sex in ASD. HTR2C and BDNF polymorphisms were correlated with severity.
Sener et al. [20]	2016	CC2D1A HTR1A	Turkey	44 patients 27 controls	Expression was associated with ASD patients.
Hamza et al. [21]	2016	GABRB3	Iraq	40 patients 25 controls	Polymorphisms were correlated with sex in ASD.
Hosseinpour et al. [22]	2017	NRP2	Iran	50 patients 70 controls	Polymorphisms were correlated with the risk of ASD.
Yuksel et al. [23]	2016	OXTR	Turkey	27 patients 39 controls	OXTR promoter methylation was associated with ASD patients.
Ocakoğlu et al. [24]	2018	OXTR	Turkey	100 patients	Polymorphisms were correlated with severity of autism spectrum disorder.
Salem et al. [25]	2013	MAOB	Egypt	53 patients 30 controls	Polymorphisms were correlated with sex in ASD.
Synaptic*/*neuronal activity					
Noroozi et al. [26]	2018	CYFIP1/2	Iran	30 patients 41 controls	Expression was associated with ASD patients.
Safari et al. [27]	2017	SNAP25	Iran	472 patients 524 controls	Polymorphisms were correlated with the risk of ASD.
Zare et al. [28]	2017	CNTNAP2	Iran	200 patients 260 controls	Polymorphisms were correlated with the risk of ASD.
Firouzabadi et al. [29]	2016	ACE	Iran	120 patients 120 controls	Polymorphisms were correlated with the risk of ASD.
Ghafouri-Fard et al. [30]	2020	BDNF, BACE1 BDNF-AS BACE1- AS	Iran	50 patients 50 controls	Expression was associated with ASD. BACE1-AS and BDNF expression were correlated with age in female participants.
Inflammatory factors					
Eftekharian et al. [31]	2018	TNF-α, IL-6, IL-17, and IL-2	Iran	30 patients 41 controls	Polymorphisms were correlated with autism.
Fallah et al. [32]	2020	IFNG IFNG-AS1	Iran	50 patients 50 controls	Expression was associated with ASD patients.
Safari et al. [33]	2020	HOTAIR	Iran	427 patients 430 controls	Polymorphisms were correlated with the risk of ASD.
Safari et al. [34]	2017	FOXP3	Iran	523 patients 472 controls	Polymorphisms were correlated with the risk of ASD.
Mostafa et al. [35]	2012	HLA-DRB1	Egypt	100 patients 100 controls	Polymorphisms were correlated with sex in ASD.
Amino acid metabolism					
Ajabi et al. [36]	2017	MTRR	Iran	142 patients 214 controls	Polymorphism was correlated with risk of autism.
Haghiri et al. [37]	2016	MTR	Iran	108 patients 130 controls	Polymorphism was correlated with increased risk of autism.
Jabbar et al. [38]	2018	MTR	Iraq	70 patients 30 controls	Polymorphisms were correlated with sex in ASD.
Behiry [39]	2017	CBS	Egypt	40 patients 40 controls	Polymorphisms were correlated with ASD.
Taheri et al. [40]	2019	MOCOS	Iran	406 patients 411 controls	Polymorphisms were correlated with the risk of ASD.
Nuclear receptors and GTPases					
Mobasheri et al. [41]	2019	VDR	Iran	81 patients 108 controls	Polymorphism was correlated with reduced risk of autism.
Coskun et al. [42]	2016	VDR	Turkey	237 patients 243 controls	Polymorphisms were correlated with ASD.
Sayad et al. [43]	2017	RORA	Iran	518 patients 472 controls	Polymorphisms were correlated with autism risk in dominant inheritance model.
Emamalizadeh et al. [44]	2017	RIT2	Iran	470 patients 470 controls	Polymorphisms were correlated with the risk of ASD.
Hamedani et al. [45]	2017	RIT2	Iran	532 patients 472 controls	Polymorphisms were correlated with the risk of ASD.
Transporters					
Noroozi et al .[46]	2017	VMAT1	Iran	495 patients 484 controls	Polymorphism was correlated with autism.
Kamal et al .[47]	2015	DRD4	Egypt	178 patients 128 controls	Polymorphisms were correlated with ASD.
Azzam et al. [48]	2018	SLC6A3 DRD1	Egypt	50 patients 50 controls	SLC6A3 polymorphisms were correlated with mother’s age. DRD1 polymorphisms were correlated with age in ASD.
El-Tarras et al. [49]	2012	SLC6A3 DRD1	Saudi Arabia	50 patients 50 controls	Polymorphisms were correlated with sex in ASD.
Transcriptional and epigenetic regulation					
Meguid [50]	2015	MTHFR	Egypt	24 patients and 20 of their mothers. 30 control and 42 healthy control mothers.	MTHFR polymorphisms were correlated with ASD and higher in autism children mothers.
El-Baz et al. [51]	2017	MTHFR	Egypt	31 patients 39 controls	Polymorphisms were correlated with sex in ASD.
Shawky et al. [52]	2014	MTHFR	Egypt	20 patients 20 controls	Polymorphisms were correlated with sex in ASD.
Arab and Elhawary [53]	2019	MTHFR	Saudi Arabia	112 patients 104 controls	Polymorphisms were correlated with sex in ASD.
Muftin et al. [54]	2020	MTHFR	Iraq	38 patients 22 controls	Polymorphisms were correlated with sex in ASD.
Beiranvandi et al. [55]	2020	EN2	Iran	67 patients 100 controls	Polymorphisms were correlated with the risk of ASD.
Eftekharian et al. [56]	2018	PIAS	Iran	30 patients 41 controls	Expression was associated with age in ASD patients.
DNA repair					
Dasdemir et al. [57]	2016	XRCC4	Turkey	100 patients 96 controls	Polymorphisms were correlated with ASD.

## Receptors and neurotransmitters

Dopamine D3 receptor (DRD3) is localized in limbic areas and cortical brain, which are involved in locomotion and emotional and cognitive processes, as well as endocrine functions. The rs6280 (C>T) SNP of the first *DRD3* exon results in a serine to glycine substitution (ser9Gly) in mental disorder [58]. The glycine allele carriers in hypercortisolemia cases might be more susceptible for developing hyperdopaminergic responses to stress [59]. It has been reported that the Ser9Gly (rs6280) Gly allele or Gly/Gly, Ser/Gly genotypes had significantly better responses to antipsychotics like risperidone in Iranian autistic patients [17]. Glutamate metabotropic receptor 7 (GRM7) is a G-protein coupled glutamate receptor which has spatiotemporal expression in the cerebral cortex, cerebellum, and hippocampus [18]. The rs779867 (T> G) is an intronic polymorphism of *GRM7* that is significantly associated with ASD in Chinese children [60]. Another study has demonstrated a correlation between rs779867 polymorphism and ASD in Iranian patients. The rs779867 G/G genotype and G allele were significantly more frequent in ASD patients compared to control group [18]. *HTR2A* encodes serotonin receptor 5-HT2A that can be epigenetically regulated through DNA methylation and regulate fetal brain development and adult cognitive function. *HTR2A* SNPs are associated with a number of psychiatric disorders [61]. *HTR2A* rs7997012 A > G intronic SNP is correlated with social adjustment in depressed [62]. The HTR2C is a G protein-coupled receptor (GPCR) that responds to signaling through the serotonin. The serotonin transporter encoded by *SLC6A4* transports the serotonin from synaptic spaces into presynaptic neurons. Functional *SLC6A4* rs3813034 (G2563T) polymorphism is located in the miR-16–binding site and affects the *SLC6A4* expression and function. It has been reported that there were significant associations between *SLC6A4* rs3813034, *HTR2A* rs7997012, *BDNF* rs6265, and *HTR2C* rs6318 polymorphisms and autism susceptibility among a subpopulation of Saudi subjects. Heterozygosity was significantly associated with the risk of ASD for the *HTR2C* rs6318 and *SLC6A4* rs3813034 variants. The *HTR2C* rs6318 and *BDNF* rs6265 SNPs were significantly correlated with severe cases. There were significant associations between *HTR2A* rs7997012 and rs6265A variants of *BDNF* and ASD [19]. Coiled-coil and C2 domain containing 1A (CC2D1A) regulates 5-hydroxytryptamine receptor 1A (HTR1A) expression in neuronal cells via binding to 14-bp 5′-repressor element upstream of the promoter of *HTR1A* [63]. Mutations in the CC2D1A signaling results in non-syndromic intellectual disability, ASD, and seizures [64]. It has been shown that there was a significant inverse association between *CC2D1A* and *HTR1A* gene expressions in blood sample of ASD patients compared to control subjects among a group of Turkish patients [20]. Gamma-aminobutyric acid receptor subunit beta-3 (GABRB3) is involved in pathogenesis of some diseases such as Angelman syndrome, Prader-Willi syndrome, epilepsy, and autism [65]. It has been reported that the *GABRB3* gene polymorphism was significantly associated with autism among Iraqi patients [21]. Neuropilins (NRPs) are non-tyrosine kinase cell surface glycoproteins which function in various types of signaling pathways. NRPs identified as coreceptors for vascular endothelial growth factor (VEGF) and class III semaphorins. Many physiological processes such as cardiovascular, neuronal development, angiogenesis, and lymphangiogenesis are associated with neuropilin glycoproteins [66]. It has been suggested that the rs849563 (T> G) polymorphism located at exon 10 of neuropilin-2 can be as risk factor for ASD among Iranian patients [22]. The OXTR is a GPCR of oxytocin that activates a set of signaling cascades, including the MAPK, PKC, PLC, or CaMK pathways [67]. OXTR and the oxytocin signaling pathway have important roles in the etiology of autism [68]. It has been reported that there was a high frequency of *OXTR* promoter hypomethylation in ASD compared with healthy control in a sample of Turkish autistic children [23]. Another study also reported a significant correlation between *OXTR* -rs237902 polymorphism and severity of autism in a subpopulation of Turkish subjects. The GA and AA genotype (GA/AA) carriers of rs237902 had more severe pathological symptoms compared with GG genotype carriers in males [24]. MAOB belonged to the monoamine oxidases involved in dopamine and serotonin oxidation. It is located in the mitochondrial outer membrane which has critical roles in neuroactive and vasoactive amine metabolisms in peripheral tissues and central nervous system [69]. It has been observed that there was a correlation between *MAOB* rs1799836 and autism among a group of Egyptian cases in which the G allele frequency was significantly more frequent in patients compared with healthy controls. Plasma MAOB activity was also significantly reduced in males with G allele compared with A allele carriers [25].

## Synaptic/neuronal activity

Cytoplasmic FMR1-interacting proteins 1/2 (CYFIP1/CYFIP2) are components of the WAVE regulatory complex (WRC) which are critical regulators of actin polymerization that is involved in spinal morphology and presynaptic activity. *CYFIP1/CYFIP2* abnormalities have been observed in neurodevelopmental disorders [70, 71]. There were significant *CYFIP1/2* upregulations in autism patients compared with healthy subjects among a subpopulation of Iranian patients. These genes might be potential biomarkers for ASD diagnosis [26]. Synaptosomal-associated protein 25 (SNAP-25) has an important role in nerve terminal plasticity [27]. The rs3746544 G>A is located in the 3′-untranslated region (3′-UTR) of *SNAP-25* associated with attention-deficit hyperactivity disorder [72]. It has been found that there was a correlation between the rs3746544 *SNAP25* and ASD patients in a sample of Iranian cases [27]. Contactin-associated protein 2 (CNTNAP2) as a member of the neurexin superfamily is a presynaptic cell adhesion protein involved in neuron connections at synapses. It is mainly expressed in the brain and spinal cord. CNTNAP2 can be a genetic risk factor of ASD and related neurodevelopmental disorders [73]. It has been observed that rs7794745 *CNTNAP2* was correlated with ASD susceptibility in Iranian population [28]. Angiotensin-converting enzyme (ACE) is an enzyme involved in regulation of blood pressure through conversion of the inactive angiotensin I to the active peptide hormone angiotensin II. Angiotensin II has a pivotal role in blood pressure regulation and fluid-electrolyte balance [74]. ACE has an important function in neurokinin degeneration which is a family of neurotransmitters involved in regulation of behavior and memory [29]. It has been reported that the rs4343, rs4291, and also *ACE* I/D polymorphism affected the activity of this enzyme and the level of Ang II [75, 76]. The rs4343 (G >A) *ACE* polymorphism is involved in hypertension, left ventricular hypertrophy, migraine, and coronary artery diseases [77]. Insertion/deletion (I/D) *ACE* polymorphism was associated with cardiovascular disease in which the D allele increased risk of cardiovascular disease. I/D polymorphism were also linked with blood pressure [78]. It has been reported that the *ACE* genetic variants were associated with autism in a group of Iranian subjects in which G allele of rs4343 was associated with ASD risk. Both DD genotype of ACE I/D and the D allele were also significantly correlated with ASD. Moreover, there was an association between rs4291 and autism among Iranian autistic patients [29]. The BACE1 is a transmembrane protease involved in amyloid precursor protein (APP) cleavage which is a regulator of synapse formation. Amyloid beta peptides constitute the amyloid beta plaques in Alzheimer’s disease (AD) pathogenesis [79, 80]. The neurotrophin brain-derived neurotrophic factor (BDNF) belongs to the nerve growth factor family that functions in development and maintenance of normal brain. It controls the survival and maturation of neurons through tropomyosin receptor kinase B (TrkB) activation. BDNF–TrkB binding triggers the PI3K/AKT signaling pathway that affects various phases of synaptic development. It has been reported that there was an association between BDNF and pathogenesis of psychiatric disorders [81]. The BDNF, BACE1, and their antisenses were significantly upregulated in Iranian autistic patients compared with matched healthy subjects. Therefore, the BDNF, BACE1, and their antisenses may have role in the pathogenesis of ASD and might be as putative markers for ASD [30]. The PI3K/AKT is reported as a critical pathway in regulation of polarity acquisition and axon branching. It is also involved in regulation of nerve cell migration to the cortical plate [82]. PTEN is a negative regulator of PI3K/AKT pathway through PI3K suppression that can also be regulated by various factors. WWP1 is an E3 ubiquitin-protein ligase involved in deactivation of PTEN that results in activation of PI3K/AKT signaling pathway. It has been observed that there was a correlation between the WWP1 germline variations and normocephalic ASD [12]. Therefore, it seems that the PI3K/AKT signaling has a pivotal role during ASD progression via aberrant WWP1/PTEN and BDNF/TrkB axis [12, 30].

## Inflammatory factors

Tumor necrosis factor (TNF-α) is an inflammatory cytokine produced by macrophages and monocytes. It binds to its receptors during acute inflammation to mediate cell proliferation, differentiation, and apoptosis [78]. IL-6 is produced in response to infectious lesion leading to inflammatory response, hematopoiesis, and immune reactions. IL-6 also functions in the maturation of B cells [83].IL-17 is secreted by CD4 Th17 and CD8 Tc17 cells to induce G-CSF, CXCL1, and CXCL2 productions [84]. IL-2 is a critical cytokine for proliferation of T and B lymphocytes. It has been observed that there were significant increased levels of TNF-α, IL-6, and IL-17 and decrease levels of IL-2 in ASD patients compared with healthy subjects among a subpopulation of Iranian autism patients [31]. Th17 cells are proinflammatory cells that involve in immunity against several extracellular pathogens. They require IL-6, TGF-β, and IL-21 for their differentiation [85]. Regulatory T cells (Tregs) and suppressor T cells belonged to a distinct T cell subset that regulates immune response. CD4, FOXP3, and CD25 are biomarkers of Tregs [86]. Interferon-γ is a soluble cytokine that functions in the immune response. It is a predominant inducer of indoleamine dioxygenase (IDO) that converts tryptophan to kynurenine in kynurenine pathway [87]. *IFNG-AS1* plays an important role in regulation of *IFNG* expression in human CD4^+^ T cells [88]. It has been shown that there was significant *IFNG* upregulation and *IFNG-AS1* downregulation in Iranian children with ASD compared with controls. The male autism children had also higher levels of *IFNG* expression compared with healthy subjects. Moreover, there was a significant direct association between *IFNG-AS1* expression level and age in ASD group [32]. The HOTAIR is a lncRNA associated with immune responses through downregulation of the NF-κB-related cytokines in macrophages [89]. The rs12826786 C>T as functional polymorphism is located in the promoter region of HOTAIR that is associated with breast cancer among Turkish patient [90]. It has been demonstrated that there was a significant association between rs12826786 in *HOTAIR* and risk of ASD in Iranian children autism patients [33]. FOXP3 is a pivotal regulator of T reg cell development and function [91]. The rs2232365 polymorphism in the promoter sequence of *FOXP3* is associated with multiple sclerosis [92], acute coronary syndrome [91], and recurrent spontaneous abortion [93]. It has been observed that there was a significant association between *FOXP3* rs2232365-G allele and ASD risk among Iranian subjects [34]. HLA-DRB1 belongs to HC class II that plays a critical role in the immune system. It has been reported that there were significant associations between HLA-DRB1*03 and HLA-DRB1*11 variants and autism among Saudi cases [35].

## Amino acid metabolism

Methionine synthase reductase (MTRR) is a family of electron transferases and plays a role in folate-dependent homocysteine/methionine metabolism which regenerate methionine synthase (MTR) to active form. Other enzymes of the homocysteine metabolic pathway are Methylenetetrahydrofolate Reductase (MTHFR) and Thymidylate Synthase (TS). MTRR is involved in reduction of MTR-cob (II) alamin to MTR-cob (I) alamin, and transform 5-methyltetrahydrofolate to tetrahydrofolate [94, 95]. The *MTRR* 66A > G (rs1801394) polymorphism changes an isoleucine into a methionine (Ile22Met) leading to the reduced enzyme activity [96]. It has been found that the rs1801394 was a risk factor in some cancers [36]. Association between rs1801394 and drug response has been also reported in some disease such as rheumatoid arthritis [97] and childhood acute lymphoblastic leukemia [98]. It has been demonstrated that there was a correlation between *MTRR* A66G polymorphism and autism in a sub population of Iranian patients. GG was higher in autistic children than controls. The result showed that the G allele might increase risk of autism [36]. Methionine synthase is involved in methyl transfer from N5-methyl-5,6,7,8-tetrahydrofolate to l-homocysteine in the final step of methionine regeneration [99]. The *MTR* A2756G (rs1805087) polymorphism changes an aspartic acid to glycine (D919G) that results in reduced enzyme activity. There was a correlation between rs1805087 SNP and *DNMT1* methylation levels in cancer cells [100]. Double heterozygosity for *MTR* 2756 AG/*MTRR* 66 AG was a significant risk factor of Down syndrome [101]. It has been shown that there was a significant correlation between G allele of *MTR* A2756G polymorphism and elevated ASD susceptibility in Northern Iranian population. It could be used as a useful molecular biomarker to predict genetic susceptibility for autism among Northern Iranian patients. The GG was more frequent in autistic children than controls [37]. Another study has shown that there was significant correlation between G allele of *MTR* A2756G and increased ASD risk in Iraqi patients [38]. Cystathionine beta synthase (CBS) regulates the homocysteine metabolism through homocysteine to cystathionine transformation. It has also a critical role in H_2_S biosynthesis which contributes to several process such as cellular energetics, DNA methylation, and protein modification [102]. There was a correlation between *CBS* (C699T) polymorphism and autism in which the T allele was significantly more frequent among Egyptian autism cases compared with controls [39]. The rs594445 C>A polymorphism in *MOCOS*, His703Asn, might modify enzyme activity that lead to reduce metabolic capacity of xanthine dehydrogenase (XDH) and aldehyde oxidase 1 (AOX1) that are important for purine metabolism. MOCOS activate XDH and AOX1 by sulfurating the molybdenum cofactor [103]. It has been reported that the A allele of rs594445 polymorphism in *MOCOS* was markedly associated with risk of autism in Iranian ASD cases compared with controls [40].

## Nuclear receptors and GTPases

Vitamin D is a steroid hormone that modulates expression of various target genes via the vitamin D receptor (VDR) during neural development and antioxidant mechanisms [104]. Vitamin D3 deficiency during pregnancy and early childhood might be a risk factor for the progression of childhood autism [105]. Genetic polymorphism in *VDR* can be associated with ASD symptoms by influencing the vitamin D3 metabolism [105]. TaqI (rs731236, T>C) polymorphism as a silent mutation alters the VDR protein structure [106]. *VDR* TaqI polymorphisms were identified in some diseases such as renal diseases, cancer, nephrolithiasis, and diabetes [105]. It has been found that TaqI (rs731236) variants can be the protective risk factors of ASD occurrence in children among Iranian subjects [41]. FokI, TaqI, and BsmI variants were also significantly associated with ASD in a sample of Turkish subjects [42]. The retinoic acid-related orphan receptors alpha (RORA) is a member of the NR1 nuclear receptors which are ligand-dependent transcription factors. RORA is involved in immunity, differentiation, and embryogenesis. There are associations between RORα expression and many cancers such as colorectal and breast carcinoma [107]. The *RORA* (rs4774388 C>T) is a functional polymorphism located in chromosome 15. It has been reported that there was a correlation between rs4774388 SNP and *IFN-β* response in multiple sclerosis patients [108]. There was also an association between rs4774388 SNP and susceptibility to bipolar disorder [108]. It has been shown that there was a correlation between T allele of *RORA* rs4774388 and ASD patients in a subpopulation of Iranian patients. The rs4774388-TT genotype was also significantly more frequent in patients compared with controls which contributed with autism risk in dominant state [43]. RIT2 belongs to the RAS superfamily of small GTPases involved in many important cellular pathways. The rs16976358 T>C *RIT2* polymorphism is associated with *RIT2* expression and function in autism, schizophrenia, and bipolar disorder [44]. It has been reported that the rs16976358 variant can be a risk factor of ASD among Iranian patients. Higher frequencies of rs16976358-C allele have been detected in healthy control compared with ASD. The rs16976358 CC and rs4130047 CC genotypes were also associated with ASD in recessive inheritance model. Moreover, the C/T haplotype block (rs16976358/ rs4130047) was correlated with ASD [45].

## Transporters

Vesicular monoamine transporter 1 (VMAT1) is an integral membrane protein in synaptic vesicles, which serves to accumulate cytosolic monoamines into synaptic vesicles. It is a target of reserpine and tetrabenazine [109]. There is a correlation between rs1390938 G/A (Thr136Ile) polymorphism and presynaptic transport of monoamines [110]. Moreover, it is associated with anxiety, affective, and alcohol addiction disorders [110]. It has been demonstrated that the rs1390938 G/A genotypes of *VMAT1* polymorphisms were significantly correlated with ASD risk in the Iranian population. The rs1390938-G allele was significantly more frequent in ASD compared with healthy control, and AA genotype was protective in dominant and recessive models. Moreover, the CATT and CATG haplotypes (rs2270637, rs1390938, rs2279709, and rs2270641 respectively) were protective against ASD [46]. The human dopamine receptor D4 (DRD4) gene is located on 11p15.5 chromosome. It is associated with psychiatric disorders and autonomic nervous system dysfunction through adenylyl cyclase inhibition [111]. It has been demonstrated that there was associated between exon III 48 bp VNTR of the *DRD4* and autism which might be a risk factor for ASD [47]. The dopamine transporter DAT (SLC6A3) gene affects neuronal networks in working memory and episodic memory [112]. It has been reported that there was a correlation between *DRD1* rs4532 polymorphism and autism among Egyptian population. The mother’s age at conception was also correlated with rs2550936 SNP at *SLC6A3* [48]. Similarly, there was a significant association between rs2550936 A/C polymorphism of *SLC6A3* and rs4532 A/G polymorphism of *DRD1* with ASD in which the GA genotype of *DRD1* and CA genotype of *SLC6A3* can be risk factor of ASD in a subpopulation of Saudi patients [49].

## Transcriptional and epigenetic regulation

MTHFR catalyzes the conversion of 5,10-methylenetetrahydrofolate to 5-methyltetrahydrofolate that has a pivotal role in DNA methylation and epigenetic regulation [113]. Epigenetic changes are important molecular alterations associated with a variety of human disorders [114–116]. It has been reported that there was a correlation between *MTHFR* A1298C polymorphism and increased risk of ASD among Egyptian cases. Genotype frequency of *MTHFR* 1298 AC/CC was significantly higher in ASD compared with healthy controls. *MTHFR* 1298 AC was also significantly more frequent in autistic children mothers in comparison with control mothers [50]. Another study showed that there were significant associations between C677T and A1298C polymorphic genotypes and autism susceptibility among Egyptian patients [51, 52]. The 677C>T rs1801133 and 1298A>C rs1801131 were also significantly associated with autism risk in Saudi subjects [53]. A *MTHFR* polymorphism assessment among Iraqi autistic children has shown that there was a significant association between g. 16,505 del C and autism susceptibility [54]. EN2 is a homeobox transcription factor involved in regulation of midbrain and cerebellum development. It has been demonstrated that there was a significant association between rs1861972 polymorphism and autism risk in a sample of Iranian cases [55]. The protein inhibitor of activated STAT (PIAS) functions as an E3 SUMO-protein ligase. The PIAS family of proteins include PIAS1, PIAS2 (PIASx), PIAAS3, and PIAS4 (PIASy). PIAS1 is known as a STAT1-interacting protein which inhibits the STAT1-mediated transcriptional activation [117]. It has been revealed that there was a significant correlation between the levels of PIASx expression and age among Iranian ASD patients [56].

## Mitochondrial DNA and DNA repair

Defects in the mitochondrial genome are frequent in autistic patients [118, 119]. The reduced nicotinamide adenine dinucleotide (NADH) oxidase activity in lymphocytic mitochondria was lower, and plasma pyruvate levels were higher in autism children than healthy children [120]. The G8363A transition on tRNALys was associated with enhanced risk of autism [121]. It has been observed that there were significant associations between 16126T>C, 14569G>A, and 1811A>G mitochondrial polymorphisms and autism susceptibility in a sample of Iranian cases [122]. XRCC4 has a pivotal role in DNA double-strand break repair [123]. DNA ligase IV-XRCC4 complex is critical for double-strand break repair and completion of V (D) J recombination in immune cells. XRCC4 mediates the binding of DNA ligase IV to DNA [124]. It has been reported that there was a correlation between *XRCC4* variants and ASD risk among a subpopulation of Turkish patients in which the *XRCC4*-1394 T/G+G/G genotypes were more frequent in patients compared with healthy subjects [57].

## Conclusions

ASD is a heterogeneous neurological disorder with diverse clinical manifestations that mainly result in social problems in patients. Therefore, it is really essential to clarify the genetic and molecular biology of ASD to introduce novel effective diagnostic and therapeutic options. Genetic aberrant of receptors and neurotransmitters is the main genetic risk factors of ASD progression among Middle East population. Regarding the various reported genes in these countries, it is not possible to introduce an efficient panel marker for all of these countries. However, assessment of MTHFR and DRD gene alterations can be suggested for the ASD screening among Arab population. Since, the reported genes among Iranians were different from the Turkish and Arab populations, a panel of five markers (based on sample size assessments) including GRM7, SNAP25, FOXP3, RORA, and RIT2 can also be suggested for the ASD screening among Iranian population. This review clarifies the genetic and molecular biology of ASD to introduce a population-based panel of genetic markers for the early ASD management and detection among Middle East population.

## Data Availability

The datasets used and/or analyzed during the current study are available from the corresponding author on reasonable request.

## Abbreviations

ASD: Autism spectrum disorder
DRD3: Dopamine D3 receptor
GRM7: Glutamate metabotropic receptor 7
GPCR: G protein-coupled receptor
CC2D1A: Coiled-coil and C2 domain containing 1A
HTR1A: 5-Hydroxytryptamine receptor 1A
GABRB3: Gamma-aminobutyric acid receptor subunit beta-3
NRPs: Neuropilins
VEGF: Vascular endothelial growth factor
CYFIP1/CYFIP2: Cytoplasmic FMR1-interacting proteins 1/2
WRC: WAVE regulatory complex
SNAP-25: Synaptosomal-associated protein 25
ADHD: Attention-deficit hyperactivity disorder
CNTNAP2: Contactin-associated protein 2
ACE: Angiotensin-converting enzyme
APP: Amyloid precursor protein
AD: Alzheimer’s disease
BDNF: Brain-derived neurotrophic factor
TrkB: Tropomycin receptor kinase B
TNF-α: Tumor necrosis factor
G-CSF: Granulocyte colony-stimulating factor
Tregs: Regulatory T cells
MTRR: Methionine synthase reductase
MTR: Methionine synthase
MTHFR: Methylenetetrahydrofolate reductase
TS: Thymidylate synthase
ZDH: Xanthine dehydrogenase
AOX1: Aldehyde oxidase 1
CBS: Cystathionine beta synthase
VDR: Vitamin D receptor
RORA: Retinoic acid-related orphan receptor alpha
VMAT1: Vesicular monoamine transporter 1
DRD4: Dopamine receptor D4
PIAS: Protein inhibitor of activated STAT
NADH: Nicotinamide adenine dinucleotide

## References

[1] Hashem S (2020). Genetics of structural and functional brain changes in autism spectrum disorder. Transl Psychiatry.

[2] Toma C (2020). Genetic variation across phenotypic severity of autism. Trends Genet.

[3] Kemper TL, Bauman ML (2002). Neuropathology of infantile autism. Mol Psychiatry.

[4] Amaral DG, Schumann CM, Nordahl CW (2008). Neuroanatomy of autism. Trends Neurosci.

[5] Xu G, Strathearn L, Liu B, Bao W (2018). Prevalence of autism spectrum disorder among US children and adolescents, 2014-2016. Jama.

[6] Qiu S, Lu Y, Li Y, Shi J, Cui H, Gu Y, Li Y, Zhong W, Zhu X, Liu Y, Cheng Y, Liu Y, Qiao Y (2020). Prevalence of autism spectrum disorder in Asia: a systematic review and meta-analysis. Psychiatry Res.

[7] Lyall K, Schmidt RJ, Hertz-Picciotto I (2014). Maternal lifestyle and environmental risk factors for autism spectrum disorders. Int J Epidemiol.

[8] Reichenberg A, Gross R, Weiser M, Bresnahan M, Silverman J, Harlap S, Rabinowitz J, Shulman C, Malaspina D, Lubin G, Knobler HY, Davidson M, Susser E (2006). Advancing paternal age and autism. Arch Gen Psychiatry.

[9] Genovese A, Butler MG (2020). Clinical assessment, genetics, and treatment approaches in autism spectrum disorder (ASD). Int J Mol Sci.

[10] Geschwind DH, State MW (2015). Gene hunting in autism spectrum disorder: on the path to precision medicine. Lancet Neurol.

[11] Waye MM, Cheng HY (2018). Genetics and epigenetics of autism: a review. Psychiatry Clin Neurosci.

[12] Novelli G, Novelli A, Borgiani P, Cocciadiferro D, Biancolella M, Agolini E, Pietrosanto M, Casalone R, Helmer-Citterich M, Giardina E, Jain SK, Wei W, Eng C, Pandolfi PP (2020). WWP1 germline variants are associated with normocephalic autism spectrum disorder. Cell Death Dis.

[13] Tremblay MW, Jiang YH (2019). DNA methylation and susceptibility to autism spectrum disorder. Annu Rev Med.

[14] Molloy C, Keddache M, Martin LJ (2005). Evidence for linkage on 21q and 7q in a subset of autism characterized by developmental regression. Mol Psychiatry.

[15] Excellence, N.I.f.C (2012). Autism: recognition, referral, diagnosis and management of adults on the autism spectrum. Natl Inst Health Care Excell.

[16] Madden JM, Lakoma MD, Lynch FL, Rusinak D, Owen-Smith AA, Coleman KJ, Quinn VP, Yau VM, Qian YX, Croen LA (2017). Psychotropic medication use among insured children with autism spectrum disorder. J Autism Dev Disord.

[17] Firouzabadi N, Nazariat A, Zomorodian K (2017). DRD3 Ser9Gly polymorphism and its influence on risperidone response in Autistic Children. J Pharm Pharm Sci.

[18] Noroozi R, Taheri M, Movafagh A, Mirfakhraie R, Solgi G, Sayad A, Mazdeh M, Darvish H (2016). Glutamate receptor, metabotropic 7 (GRM7) gene variations and susceptibility to autism: a case-control study. Autism Res.

[19] Elhawary NA, Tayeb MT, Sindi IA, Qutub N, Rashad M, Mufti A, Arab AH, Khogeer A, Elhawary EN, Dannoun A, Bogari N (2019). Genetic biomarkers predict susceptibility to autism spectrum disorder through interactive models of inheritance in a Saudi community. Cogent Biol.

[20] Sener EF, Cıkılı Uytun M, Korkmaz Bayramov K, Zararsiz G, Oztop DB, Canatan H, Ozkul Y (2016). The roles of CC2D1A and HTR1A gene expressions in autism spectrum disorders. Metab Brain Dis.

[21] Hamza A, Al-Kazaz A, Muhsin A (2016). Identification of genetic mutations associated with autism in GABRB3 gene in Iraqi autistic patients.

[22] Hosseinpour M, Mashayekhi F, Bidabadi E, Salehi Z (2017). Neuropilin-2 rs849563 gene variations and susceptibility to autism in Iranian population: a case-control study. Metab Brain Dis.

[23] Elagoz Yuksel M, Yuceturk B, Karatas OF, Ozen M, Dogangun B (2016). The altered promoter methylation of oxytocin receptor gene in autism. J Neurogenet.

[24] Ocakoğlu FT, Köse S, Özbaran B, Onay H (2018). The oxytocin receptor gene polymorphism -rs237902- is associated with the severity of autism spectrum disorder: a pilot study. Asian J Psychiatr.

[25] Salem AM (2013). Genetic variants of neurotransmitter-related genes and miRNAs in Egyptian autistic patients. Sci World J.

[26] Noroozi R, Omrani MD, Sayad A, Taheri M, Ghafouri-Fard S (2018). Cytoplasmic FMRP interacting protein 1/2 (CYFIP1/2) expression analysis in autism. Metab Brain Dis.

[27] Safari MR (2017). Synaptosome-associated protein 25 (SNAP25) gene association analysis revealed risk variants for ASD, in Iranian population. J Mol Neurosci.

[28] Zare S, Mashayekhi F, Bidabadi E (2017). The association of CNTNAP2 rs7794745 gene polymorphism and autism in Iranian population. J Clin Neurosci.

[29] Firouzabadi N, Ghazanfari N, Alavi Shoushtari A, Erfani N, Fathi F, Bazrafkan M, Bahramali E (2016). Genetic variants of angiotensin-converting enzyme are linked to autism: a case-control study. PLoS One.

[30] Ghafouri-Fard S, Namvar A, Arsang-Jang S, Komaki A, Taheri M (2020). Expression analysis of BDNF, BACE1, and their natural occurring antisenses in autistic patients. J Mol Neurosci.

[31] Eftekharian MM, Ghafouri-Fard S, Noroozi R, Omrani MD, Arsang-jang S, Ganji M, Gharzi V, Noroozi H, Komaki A, Mazdeh M, Taheri M (2018). Cytokine profile in autistic patients. Cytokine.

[32] Fallah H, Sayad A, Ranjbaran F, Talebian F, Ghafouri-Fard S, Taheri M (2020). IFNG/IFNG-AS1 expression level balance: implications for autism spectrum disorder. Metab Brain Dis.

[33] Safari M, Noroozi R, Taheri M, Ghafouri-Fard S (2020). The rs12826786 in HOTAIR lncRNA is associated with risk of autism spectrum disorder. J Mol Neurosci.

[34] Safari MR, Ghafouri-Fard S, Noroozi R, Sayad A, Omrani MD, Komaki A, Eftekharian MM, Taheri M (2017). FOXP3 gene variations and susceptibility to autism: a case–control study. Gene.

[35] Mostafa GA, Shehab AA, Al-Ayadhi LY (2013). The link between some alleles on human leukocyte antigen system and autism in children. J Neuroimmunol.

[36] Ajabi S, Mashayekhi F, Bidabadi E. A study of MTRR 66A>G gene polymorphism in patients with autism from northern Iran. Neurology Asia. 2017;22(1).

[37] Haghiri R, Mashayekhi F, Bidabadi E, Salehi Z (2016). Analysis of methionine synthase (rs1805087) gene polymorphism in autism patients in Northern Iran. Acta Neurobiol Exp.

[38] Jabbar AR, Jebor MA. Study of polymorphism in methionine synthase gene by RFLP-PCR in middle Euphrates region of Iraq. J Pharm Sci Res. 2018;10(12):3219.

[39] Behiry E (2017). Association of cystathionine beta synthase gene polymorphism with cognitive disorders in autistic children. J Innov Pharmaceutical Biol Res.

[40] Taheri M, Noroozi R, Aghaei K, Omrani MD, Ghafouri-Fard S (2020). The rs594445 in MOCOS gene is associated with risk of autism spectrum disorder. Metab Brain Dis.

[41] Mobasheri L, Moossavi SZ, Esmaeili A, Mohammadoo-khorasani M, Sarab GA (2020). Association between vitamin D receptor gene FokI and TaqI variants with autism spectrum disorder predisposition in Iranian population. Gene.

[42] Coşkun S, Şimşek Ş, Camkurt MA, Çim A, Çelik SB (2016). Association of polymorphisms in the vitamin D receptor gene and serum 25-hydroxyvitamin D levels in children with autism spectrum disorder. Gene.

[43] Sayad A, Noroozi R, Omrani MD, Taheri M, Ghafouri-Fard S (2017). Retinoic acid-related orphan receptor alpha (RORA) variants are associated with autism spectrum disorder. Metab Brain Dis.

[44] Emamalizadeh B, Jamshidi J, Movafagh A, Ohadi M, khaniani MS, Kazeminasab S, Biglarian A, Taghavi S, Motallebi M, Fazeli A, Ahmadifard A, Shahidi GA, Petramfar P, Shahmohammadibeni N, Dadkhah T, Khademi E, Tafakhori A, Khaligh A, Safaralizadeh T, Kowsari A, Mirabzadeh A, Zarneh AES, Khorrami M, Shokraeian P, Banavandi MJS, Lima BS, Andarva M, Alehabib E, Atakhorrami M, Darvish H (2017). RIT2 polymorphisms: is there a differential association?. Mol Neurobiol.

[45] Hamedani SY, Gharesouran J, Noroozi R, Sayad A, Omrani MD, Mir A, Afjeh SSA, Toghi M, Manoochehrabadi S, Ghafouri-Fard S, Taheri M (2017). Ras-like without CAAX 2 (RIT2): a susceptibility gene for autism spectrum disorder. Metab Brain Dis.

[46] Noroozi R, Ghafouri-Fard S, Omrani MD, Habibi M, Sayad A, Taheri M (2017). Association study of the vesicular monoamine transporter 1 (VMAT1) gene with autism in an Iranian population. Gene.

[47] Kamal M, Nady G, Abushady A, Khalil M. Association of dopamine D4 receptor gene variants with autism. Int J Res Med Sci. 2015:2658–63. 10.18203/2320-6012.ijrms20150809.

[48] Aziz Azzam A, Rasheed Bahgat DM, Hosny Shahin RM, Azme Nasralla RM (2018). Association study between polymorphisms of dopamine transporter gene (SLC6A3), dopamine D1 receptor gene (DRD1), and autism. J Med Sci Res.

[49] El-Tarras AE, Awad NS, Mitwaly N, Alsulaimani AA, Said MM. Association between polymorphisms of SLC6A3 and DRD1 genes and autism among Saudi Arabia Taif population using PCR-restriction fragment length polymorphism (PCR-RFLP). Afr J Biotechnol. 2012;11(54):11665–70.

[50] Meguid N, Khalil R, Gebril O, El-Fishawy P. Evaluation of MTHFR genetic polymorphism as a risk factor in Egyptian autistic children and mothers. J Psychiatry. 2015;18(1).

[51] El-Baz F (2017). Study of the C677T and 1298AC polymorphic genotypes of MTHFR gene in autism spectrum disorder. Electron Physician.

[52] Shawky R (2014). Study of genotype–phenotype correlation of methylene tetrahydrofolate reductase (MTHFR) gene polymorphisms in a sample of Egyptian autistic children. Egypt J Med Hum Genetics.

[53] Arab AH, Elhawary NA (2019). Methylenetetrahydrofolate reductase gene variants confer potential vulnerability to autism spectrum disorder in a Saudi community. Neuropsychiatr Dis Treat.

[54] Muftin NQ, Jubair S, Hadi SM (2020). Identification of MTHFR genetic polymorphism in Iraqi autistic children. Gene Rep.

[55] Beiranvandi F, Akouchekian M, Javadi GR, Darvish H (2020). The association of CNTNAP2 rs2710102 and ENGRAILED-2 rs1861972 genes polymorphism and autism in Iranian population. Meta Gene.

[56] Eftekharian MM, Noroozi R, Omrani MD, Arsang-Jang S, Komaki A, Taheri M, Ghafouri-Fard S (2018). Expression analysis of protein inhibitor of activated STAT (PIAS) genes in autistic patients. Adv Neuroimmune Biol.

[57] Dasdemir S (2016). DNA repair gene XPD Asp312Asn and XRCC4 G-1394T polymorphisms and the risk of autism spectrum disorder. Cell Mol Biol (Noisy-le-Grand, France).

[58] Lannfelt L, Sokoloff P, Martres MP, Pilon C, Giros B, Jönsson E, Sedvall G, Schwartz JC (1992). Amino acid substitution in the dopamine D_3_ receptor as a useful polymorphism for investigating psychiatric disorders. Psychiatr Genet.

[59] Savitz J, Hodgkinson CA, Martin-Soelch C, Shen PH, Szczepanik J, Nugent A, Herscovitch P, Grace AA, Goldman D, Drevets WC (2013). The functional DRD3 Ser9Gly polymorphism (rs6280) is pleiotropic, affecting reward as well as movement. PLoS One.

[60] Yang Y, Pan C (2013). Role of metabotropic glutamate receptor 7 in autism spectrum disorders: a pilot study. Life Sci.

[61] Paquette AG, Marsit CJ (2014). The developmental basis of epigenetic regulation of HTR2A and psychiatric outcomes. J Cell Biochem.

[62] Antypa N, Calati R, Souery D, Pellegrini S, Sentissi O, Amital D, Moser U, Montgomery S, Kasper S, Zohar J, de Ronchi D, Mendlewicz J, Serretti A (2013). Variation in the HTR1A and HTR2A genes and social adjustment in depressed patients. J Affect Disord.

[63] Vahid-Ansari F, Daigle M, Manzini MC, Tanaka KF, Hen R, Geddes SD, Béïque JC, James J, Merali Z, Albert PR (2017). Abrogated Freud-1/Cc2d1a repression of 5-HT1A autoreceptors induce fluoxetine-resistant anxiety/depression-Like behavior. J Neurosci.

[64] Zamarbide M, Oaks AW, Pond HL, Adelman JS, Manzini MC (2018). Loss of the intellectual disability and autism gene Cc2d1a and its homolog Cc2d1b differentially affect spatial memory, anxiety, and hyperactivity. Front Genet.

[65] Delahanty RJ, Zhang Y, Bichell TJ, Shen W, Verdier K, Macdonald RL, Xu L, Boyd K, Williams J, Kang JQ (2016). Beyond epilepsy and autism: disruption of GABRB3 causes ocular hypopigmentation. Cell Rep.

[66] Roy S, Bag AK, Singh RK, Talmadge JE, Batra SK, Datta K (2017). Multifaceted role of neuropilins in the immune system: potential targets for immunotherapy. Front Immunol.

[67] Jurek B, Neumann ID. The oxytocin receptor: from intracellular signaling to behavior. Physiological reviews. 2018;98(3):1805–908. 10.1152/physrev.00031.2017 29897293

[68] Gregory SG, Connelly JJ, Towers AJ, Johnson J, Biscocho D, Markunas CA, Lintas C, Abramson RK, Wright HH, Ellis P, Langford CF, Worley G, Delong GR, Murphy SK, Cuccaro ML, Persico A, Pericak-Vance MA (2009). Genomic and epigenetic evidence for oxytocin receptor deficiency in autism. BMC Med.

[69] Jakubauskiene E, Janaviciute V, Peciuliene I, Söderkvist P, Kanopka A (2012). G/A polymorphism in intronic sequence affects the processing of MAO-B gene in patients with Parkinson disease. FEBS Lett.

[70] Nakashima M, Kato M, Aoto K, Shiina M, Belal H, Mukaida S, Kumada S, Sato A, Zerem A, Lerman-Sagie T, Lev D, Leong HY, Tsurusaki Y, Mizuguchi T, Miyatake S, Miyake N, Ogata K, Saitsu H, Matsumoto N (2018). De novo hotspot variants in CYFIP2 cause early-onset epileptic encephalopathy. Ann Neurol.

[71] Zhang Y, Lee Y, Han K (2019). Neuronal function and dysfunction of CYFIP2: from actin dynamics to early infantile epileptic encephalopathy. BMB Rep.

[72] Wang C, Yang B, Fang D, Zeng H, Chen X, Peng G, Cheng Q, Liang G (2018). The impact of SNAP25 on brain functional connectivity density and working memory in ADHD. Biol Psychol.

[73] Peñagarikano O, Geschwind DH (2012). What does CNTNAP2 reveal about autism spectrum disorder?. Trends Mol Med.

[74] Fleming I (2006). Signaling by the angiotensin-converting enzyme. Circ Res.

[75] Firouzabadi N, Shafiei M, Bahramali E, Ebrahimi SA, Bakhshandeh H, Tajik N (2012). Association of angiotensin-converting enzyme (ACE) gene polymorphism with elevated serum ACE activity and major depression in an Iranian population. Psychiatry Res.

[76] Zhu X, Bouzekri N, Southam L, Cooper RS, Adeyemo A, McKenzie CA, Luke A, Chen G, Elston RC, Ward R (2001). Linkage and association analysis of angiotensin I–converting enzyme (ACE)–gene polymorphisms with ACE concentration and blood pressure. Am J Hum Genet.

[77] Abedin-Do A, Pouriamanesh S, Kamaliyan Z, Mirfakhraie R (2017). Angiotensin-converting enzyme gene rs4343 polymorphism increases susceptibility to migraine. CNS Neuroscience Ther.

[78] Idriss HT, Naismith JH (2000). TNFα and the TNF receptor superfamily: structure-function relationship(s). Microsc Res Tech.

[79] Cole S, Vassar R (2008). The basic biology of BACE1: a key therapeutic target for Alzheimer’s disease. Curr Genomics.

[80] Cole SL, Vassar R (2007). The Alzheimer’s disease β-secretase enzyme, BACE1. Mol Neurodegener.

[81] Cattaneo A, Cattane N, Begni V, Pariante CM, Riva MA (2016). The human BDNF gene: peripheral gene expression and protein levels as biomarkers for psychiatric disorders. Transl Psychiatry.

[82] Ambrozkiewicz MC, Schwark M, Kishimoto-Suga M, Borisova E, Hori K, Salazar-Lázaro A, Rusanova A, Altas B, Piepkorn L, Bessa P, Schaub T, Zhang X, Rabe T, Ripamonti S, Rosário M, Akiyama H, Jahn O, Kobayashi T, Hoshino M, Tarabykin V, Kawabe H (2018). Polarity acquisition in cortical neurons is driven by synergistic action of Sox9-regulated Wwp1 and Wwp2 E3 ubiquitin ligases and intronic miR-140. Neuron.

[83] Tanaka T, Narazaki M, Kishimoto T (2014). IL-6 in inflammation, immunity, and disease. Cold Spring Harb Perspect Biol.

[84] Kuwabara T (2017). The role of IL-17 and related cytokines in inflammatory autoimmune diseases. Mediat Inflamm.

[85] Lee GR (2018). The balance of Th17 versus Treg cells in autoimmunity. Int J Mol Sci.

[86] Goswami R, Kaplan MH. In: Galluzzi L, editor. Chapter four - STAT transcription factors in T cell control of health and disease, in International review of cell and molecular biology: Academic Press; 2017. p. 123–80. 10.1016/bs.ircmb.2016.09.012 28325211

[87] Wilson KE, Demyanovich H, Rubin LH, Wehring HJ, Kilday C, Kelly DL (2018). Relationship of interferon-γ to cognitive function in midlife women with schizophrenia. Psychiatry Q.

[88] Peng H (2015). The long noncoding RNA IFNG-AS1 promotes T helper type 1 cells response in patients with Hashimoto’s thyroiditis. Sci Rep.

[89] Obaid M, Udden SMN, Deb P, Shihabeddin N, Zaki MH, Mandal SS (2018). LncRNA HOTAIR regulates lipopolysaccharide-induced cytokine expression and inflammatory response in macrophages. Sci Rep.

[90] Bayram S, Sümbül AT, Dadaş E (2016). A functional HOTAIR rs12826786 C>T polymorphism is associated with breast cancer susceptibility and poor clinicopathological characteristics in a Turkish population: a hospital-based case–control study. Tumor Biol.

[91] Gholami M, Esfandiary A, Vatanparast M, Mirfakhraie R, Hosseini MM, Ghafouri-Fard S (2016). Genetic variants and expression study of FOXP3 gene in acute coronary syndrome in Iranian patients. Cell Biochem Funct.

[92] Gholami, M., et al., Functional genetic variants of FOXP3 and risk of multiple sclerosis. Iranian Red Crescent Medical Journal, 2016. In Press, Functional Genetic Variants of FOXP3 and Risk of Multiple Sclerosis.

[93] Wu Z (2012). Association between functional polymorphisms of Foxp3 gene and the occurrence of unexplained recurrent spontaneous abortion in a Chinese Han population. Clin Dev Immunol.

[94] Basir A (2019). Methionine synthase reductase-A66G and -C524T single nucleotide polymorphisms and prostate cancer: a case-control trial. Asian Pacific J Cancer Prev.

[95] Rai V, Yadav U, Kumar P, Yadav SK (2013). Analysis of methionine synthase reductase polymorphism (A66G) in Indian Muslim population. Indian J Hum Genetics.

[96] Olteanu H, Munson T, Banerjee R (2002). Differences in the efficiency of reductive activation of methionine synthase and exogenous electron acceptors between the common polymorphic variants of human methionine synthase reductase. Biochemistry.

[97] López-Rodríguez R, Ferreiro-Iglesias A, Lima A, Bernardes M, Pawlik A, Paradowska-Gorycka A, Świerkot J, Slezak R, Dolžan V, González-Álvaro I, Narváez J, Cáliz R, Pérez-Pampín E, Mera-Varela A, Vidal-Bralo L, Acuña Ochoa JG, Conde C, Gómez-Reino JJ, González A (2018). Replication study of polymorphisms associated with response to methotrexate in patients with rheumatoid arthritis. Sci Rep.

[98] Huang L, Tissing WJE, de Jonge R, van Zelst BD, Pieters R (2008). Polymorphisms in folate-related genes: association with side effects of high-dose methotrexate in childhood acute lymphoblastic leukemia. Leukemia.

[99] Pejchal R, Ludwig ML (2005). Cobalamin-independent methionine synthase (MetE): a face-to-face double barrel that evolved by gene duplication. PLoS Biol.

[100] Paz MF (2002). Germ-line variants in methyl-group metabolism genes and susceptibility to DNA methylation in normal tissues and human primary tumors. Cancer Res.

[101] Bosco P, Guéant-Rodriguez RM, Anello G, Barone C, Namour F, Caraci F, Romano A, Romano C, Guéant JL (2003). Methionine synthase (MTR) 2756 (A → G) polymorphism, double heterozygosity methionine synthase 2756 AG/methionine synthase reductase (MTRR) 66 AG, and elevated homocysteinemia are three risk factors for having a child with Down syndrome. Am J Med Genet A.

[102] Zhu H (2018). Cystathionine β-synthase in physiology and cancer. Biomed Res Int.

[103] Kurzawski M, Dziewanowski K, Safranow K, Drozdzik M. Polymorphism of genes involved in purine metabolism (XDH, AOX1, MOCOS) in kidney transplant recipients receiving azathioprine. Ther Drug Monit. 2012;34(3):266–74. 10.1097/FTD.0b013e31824aa681 22495427

[104] Eyles DW, Burne THJ, McGrath JJ (2013). Vitamin D, effects on brain development, adult brain function and the links between low levels of vitamin D and neuropsychiatric disease. Front Neuroendocrinol.

[105] Cieślińska A, Kostyra E, Chwała B, Moszyńska-Dumara M, Fiedorowicz E, Teodorowicz M, Savelkoul HF. Vitamin D receptor gene polymorphisms associated with childhood autism. Brain Sci. 2017;7(9):115. 10.3390/brainsci7090115 PMC5615256 28891930

[106] Uitterlinden AG, Fang Y, van Meurs JBJ, Pols HAP, van Leeuwen JPTM (2004). Genetics and biology of vitamin D receptor polymorphisms. Gene.

[107] Cook DN, Kang HS, Jetten AM (2015). Retinoic acid-related orphan receptors (RORs): regulatory functions in immunity, development, Circadian rhythm, and metabolism. Nuclear Receptor Res.

[108] Ayatollahi SA, Ghafouri-Fard S, Taheri M, Noroozi R (2020). The efficacy of interferon-beta therapy in multiple sclerosis patients: investigation of the RORA gene as a predictive biomarker. Pharmacogenomics J.

[109] Liu Y, Peter D, Merickel A, Krantz D, Finn JP, Edwards RH (1995). A molecular analysis of vesicular amine transport. Behav Brain Res.

[110] Vaht M, Kiive E, Veidebaum T, Harro J. A functional vesicular monoamine transporter 1 (VMAT1) gene variant is associated with affect and the prevalence of anxiety, affective, and alcohol use disorders in a longitudinal population-representative birth cohort study. Int J Neuropsychopharmacol. 2016;19(7). 10.1093/ijnp/pyw013 PMC4966275 26861143

[111] Ptácek R, Kuzelová H, Stefano GB (2011). Dopamine D4 receptor gene DRD4 and its association with psychiatric disorders. Med Sci Monit.

[112] Dresler T, Ehlis AC, Heinzel S, Renner TJ, Reif A, Baehne CG, Heine M, Boreatti-Hümmer A, Jacob CP, Lesch KP, Fallgatter AJ (2010). Dopamine transporter (SLC6A3) genotype impacts neurophysiological correlates of cognitive response control in an adult sample of patients with ADHD. Neuropsychopharmacology.

[113] Gales A, Masingue M, Millecamps S, Giraudier S, Grosliere L, Adam C, Salim C, Navarro V, Nadjar Y (2018). Adolescence/adult onset MTHFR deficiency may manifest as isolated and treatable distinct neuro-psychiatric syndromes. Orphanet J Rare Dis.

[114] Moghbeli M, Forghanifard MM, Sadrizadeh A, Mozaffari HM, Golmakani E, Abbaszadegan MR (2015). Role of Msi1 and MAML1 in regulation of notch signaling pathway in patients with esophageal squamous cell carcinoma. J Gastrointest Cancer.

[115] Moghbeli M, Moaven O, Memar B, Raziei HR, Aarabi A, Dadkhah E, Forghanifard MM, Manzari F, Abbaszadegan MR (2014). Role of hMLH1 and E-cadherin promoter methylation in gastric cancer progression. J Gastrointest Cancer.

[116] Moghbeli M, Rad A, Farshchian M, Taghehchian N, Gholamin M, Abbaszadegan MR (2016). Correlation between Meis1 and Msi1 in esophageal squamous cell carcinoma. J Gastrointest Cancer.

[117] Schmidt D, Müller S (2002). Members of the PIAS family act as SUMO ligases for c-Jun and p53 and repress p53 activity. Proc Natl Acad Sci U S A.

[118] Dhillon S, Hellings JA, Butler MG (2011). Genetics and mitochondrial abnormalities in autism spectrum disorders: a review. Curr Genomics.

[119] Wang Y, Picard M, Gu Z (2016). Genetic evidence for elevated pathogenicity of mitochondrial DNA heteroplasmy in autism spectrum disorder. PLoS Genet.

[120] Giulivi C, Zhang YF, Omanska-Klusek A, Ross-Inta C, Wong S, Hertz-Picciotto I, Tassone F, Pessah IN (2010). Mitochondrial dysfunction in autism. JAMA.

[121] Virgilio R, Ronchi D, Bordoni A, Fassone E, Bonato S, Donadoni C, Torgano G, Moggio M, Corti S, Bresolin N, Comi GP (2009). Mitochondrial DNA G8363A mutation in the tRNALys gene: clinical, biochemical and pathological study. J Neurol Sci.

[122] Moosavizadeh K (2013). Association of mtDNA mutation with autism in Iranian patients. Int J Pediatr.

[123] Lee K-J, Huang J, Takeda Y, Dynan WS (2000). DNA ligase IV and XRCC4 form a stable mixed tetramer that functions synergistically with other repair factors in a cell-free end-joining system. J Biol Chem.

[124] Chen L, Trujillo K, Sung P, Tomkinson AE (2000). Interactions of the DNA Ligase IV-XRCC4 complex with DNA ends and the DNA-dependent protein kinase. J Biol Chem.

